# Evaluating a dialogue-based approach to teaching about values and policy in graduate transdisciplinary environmental science programs

**DOI:** 10.1371/journal.pone.0202948

**Published:** 2018-09-04

**Authors:** Troy E. Hall, Zachary Piso, Jesse Engebretson, Michael O’Rourke

**Affiliations:** 1 Department of Forest Ecosystems and Society, Oregon State University, Corvallis, Oregon, United States of America; 2 Department of Philosophy, University of Dayton, Dayton, Ohio, United States of America; 3 Department of Philosophy, Michigan State University, East Lansing, Michigan, United States of America; 4 AgBioResearch, Michigan State University, East Lansing, Michigan, United States of America; 5 Center for Interdisciplinarity, Michigan State University, East Lansing, Michigan, United States of America; University of Toronto, Rotman School, CANADA

## Abstract

This article discusses a formal evaluation of new curricular materials and activities designed to foster understanding of three key issues–expertise, risk, and sociopolitical constraints–related to values and policy in transdisciplinary environmental science. We begin by describing the three issues, along with current thinking about the most appropriate ways to address them in the context of transdisciplinary environmental science. We then describe how we created curricular materials and activities focusing on these three issues that could be tailored for use in a wide range of graduate environmental science programs. The curriculum was adapted by instructors for use in five graduate classes at two US universities, and we used a pre-test, post-test mixed methods design to evaluate its effects on students’ ethical reasoning about values and policy. The results of this evaluation suggest that our semi-structured, dialogue-based curriculum enhances student awareness of and reasoning about values and policy in environmental research. We close with several educational recommendations for transdisciplinary environmental science programs that are grounded in our experience with this curriculum.

## Introduction

Graduate students in the environmental sciences prepare for careers where they will grapple with complex issues related to understanding environmental problems and managing natural resources [1]. Much of this work is *transdisciplinary*, in that it requires integration of perspectives from different disciplines engaged in studying environmental issues, communities who are affected by environmental problems, and policy makers charged with determining appropriate responses [2,3,4,5,6]. The term ‘transdisciplinary’ is notoriously ambiguous [3]. It is well-established that the term can be used to mean either *transformative interdisciplinarity*, where it highlights a level and type of integration that exceeds what is found in interdisciplinarity (e.g., [4]), or activity that involves the integration of insights drawn from both academic and non-academic perspectives (e.g., [5]). We mean the term in the latter sense. Further, scholars and practitioners from various disciplines and fields have identified transdisciplinary approaches as necessary to mitigate a variety of complex problems by scholars and practitioners from various disciplines and fields [6]. Because of the diverse and often contradictory nature of these perspectives, transdisciplinary efforts require that students learn how to work with public stakeholders and how to appropriately engage at the science-policy interface [7,8,9].

Success in this sort of environmental science career, then, involves cultivating what Stokols has called a *transdisciplinary orientation*, which is a combination of “collaborative values, beliefs, attitudes, and behaviors” that prepare one to interact successfully with both researchers from different disciplines and non-researchers from many communities ([10], p. 60). This type of orientation is reflected in the best practices of groups whose representatives are actively engaged in environmental work; the National Academy of Engineering, for example, recently called for the development of students’ abilities to identify relevant stakeholders and their perspectives, identify value conflicts, consider the public defensibility of actions, recognize constraints and institutional barriers, and engage in reasoned dialogue [11].

For environmental science students to cultivate a transdisciplinary orientation and become effective participants in these potentially divisive settings, environmental science graduate programs need to train students to communicate effectively with scientists from other disciplines and members of different stakeholder communities [12]. Some graduate programs have begun to address these needs by providing students with tools and skills to engage diverse stakeholders in the application of science to address environmental issues [13,14]. Principal among these tools and skills are those that enable students to understand and reason about the roles played by values (i.e., beliefs about which actions are good or bad, right or wrong) in the practices of science and policy-making. This is a form of *ethical reasoning*, which the American Association of Colleges and Universities defines as:

reasoning about right and wrong human conduct. It requires students to be able to assess their own ethical values and the social context of problems, recognize ethical issues in a variety of settings, think about how different ethical perspectives might be applied to ethical dilemmas and consider the ramifications of alternative actions. Students’ ethical self identity evolves as they practice ethical decision-making skills and learn how to describe and analyze positions on ethical issues. [15]

In transdisciplinary environmental science, students equipped with strong ethical reasoning will be reflexive about their individual and disciplinary values, sensitive to how these and other values manifest in diverse social contexts, and knowledgeable about the ramification of different policy responses in terms of stakeholders’ values [16,17,9] . However, few educational resources supporting the development of ethical reasoning in transdisciplinary environmental science currently exist [18].

In this paper, we describe the results of a study that formally evaluates a novel and newly implemented curriculum in terms of its effects on certain aspects of ethical reasoning. To properly contextualize the results of this evaluation, we also describe the new curricular materials and activities–available on-line at http://eese.msu.edu/ –which are designed to foster understanding of three key domains related to values and policy in transdisciplinary environmental research. These domains–expertise, risk, and sociopolitical constraints–involve value-laden considerations that have an ethical bearing on the formulation of and response to socio-environmental concerns. (The curriculum included a fourth domain, non-human impacts, but it was not included in the evaluation for this paper because we chose to focus on students’ reasoning about how human actors should be involved in transdisciplinary research.)

In what follows, we begin by describing the three domains, along with current thinking about the most appropriate ways to address them in socio-environmental contexts. We then describe how we created curricular materials and activities that could be tailored for use in a wide range of graduate environmental science programs. The curriculum was adapted by instructors for use in five graduate classes at two US universities, and we used a pre-test, post-test mixed methods design to evaluate its effects on students’ reasoning about values and policy. Our results suggest that the semi-structured, dialogue-based curriculum can significantly enhance students’ awareness of and the sophistication of their reasoning about values and policy in environmental research.

### Values and policy in transdisciplinary environmental science

Our interest in this paper is in graduate education in environmental science, since that is where many future environmental scientists receive their training; this is an especially good context for curricular innovation designed to enhance the transdisciplinarity of environmental science training. In fact, there are graduate programs in the environmental sciences that advocate for transdisciplinary approaches to research [8,19,20]. Transdisciplinary approaches provide the stakeholders and communities affected by issues a voice and active role in the research process from beginning to end [10]. The involvement of non-academic actors helps ensure that all forms of pertinent knowledge are brought to bear, while providing opportunities to explicitly address and reconcile different stakeholders’ values and preferences [21]. When done well, transdisciplinary research can broaden community ownership in the process and its outcomes.

So conceived, training in transdisciplinary research differs in important ways from conventional scientific training. Traditionally, students in the sciences are taught a value-free ideal of science, namely that “social, ethical, and political values should have no influence over the reasoning of scientists” ([22], p. 1). This ideal separates scientific communities from policy decisions and is predicated on the idea that scientists should not engage, as scientists, in the policy realm. When training is guided by this philosophy, students may gain in-depth knowledge about specialized disciplines, but they have few opportunities to consider how values do or should figure into research and policy processes. Recognizing this disjuncture between traditional and transdisciplinary scholarship, scholars have been calling for a reconciliation of values and scientific practice, while still maintaining the integrity of science [21].

While there are many facets of values and policy in transdisciplinary environmental science that might be addressed in graduate training, we selected three as our focus: expertise, sociopolitical constraints on research, and risk. The conduct of environmental science research and its implications for policy depend on whose expertise is considered, how the research is constrained by society, and how risks are identified and weighed, among other things. Each of these ethical considerations is imbued with values, and successful pursuit of socio-environmental goals will require identifying and negotiating those values. Given this, understanding these domains requires the ability to map and negotiate values and goals in just and equitable ways [23]. Each of the three domains is relevant–albeit in slightly different ways–at all phases of transdisciplinary research. For the purposes of this paper, we consider four research phases: conceptualizing the problem and research question, collecting relevant information, analyzing and interpreting information, and decision-making. These phases form a framework for evaluating a range of competencies that support ethical reasoning about the domains of expertise, sociopolitical constraints, and risk in the socio-environmental context.

Understanding *expertise* requires judgments about what claims qualify as knowledge and what persons are qualified to make these judgments and contribute knowledge in the research process. There are questions of expertise in all phases of research [19]. For example, stakeholders who are affected by a problem–and its responses–may be entitled to bring their own expertise to bear in defining the problem and in assessing whether potential responses to the problem are acceptable and desirable [20,16]. Scientists must also grapple with situations where there are legitimate differences of opinion about what information is relevant to a problem or even what the information means for how society should respond to the problem.

Expertise is also at stake when deciding between policy responses to a problem, raising questions about the proper role of scientists in these decisions. This issue of advocacy is hotly debated among scientists [24,25]. Some contemporary science scholars argue that where policy alternatives are controversial and/or have differential impacts on different communities, scientists should confine their influence to describing the likelihood of outcomes of different policies or actions, but they should recognize that the final decision is not resolvable by appeal to scientific evidence alone [16]. Others explore the conditions under which scientists *are* qualified to make value judgments in recommending policy responses, such as when stakeholders judge scientists to be trustworthy [26].

Related to expertise is our second domain, boundaries on research posed by society, which we call “*sociopolitical constraints*.” Apart from formal regulations and norms that govern scientific practice, researchers often face decisions about whether and how to engage in research on controversial topics. For example, they must decide whether to explore questions that are unpopular with some constituencies (such as the impacts of fracking on water quality). Similarly, they may decide to investigate unpopular policy options, such as the use of woody biomass for energy production. How the public understands scientific methods and findings can itself pose another constraint on research, insofar as the choice of methods can impact public acceptance of scientific findings. For example, researchers may face a tension between using advanced but arcane methods versus methods that are more accessible to lay audiences, but less accurate or precise. If stakeholders are not capable of understanding the techniques, data, and outcomes of science, they may not make well-informed decisions (cf. [27]).

The final curriculum domain we evaluate in this paper is “*risk*,” which concerns the magnitude and certainty of harms and benefits associated with environmental problems and responses. Like the other two domains, risk is multifaceted and comes into play in all phases of research [28]. Initially, any effort to address an environmental problem must establish the public’s role in identifying which harms should be addressed and how they should be evaluated. In many environmental problems, it is important to consider whether certain social groups are differentially exposed to harms [29]. When it comes to the decision-making phase, scientists must decide whether they have sufficient certainty in their knowledge to defend conclusions with implications for environmental policy and management [30]. They also must make choices about how to represent assumptions, uncertainties, and variability in their findings [31]. Researchers have an obligation to describe risks accurately and clearly; ultimately, though, the public must resolve questions of values, for instance, whether to adopt a risky or harmful, but inexpensive, response or a more expensive but less risky one.

### A values and policy curriculum

#### Pedagogical considerations

In this article, we describe a formal evaluation of a curriculum module designed to enhance environmental science students’ awareness of and ability to reason about the domains discussed above. To develop our curricular materials, we reviewed best practices in graduate pedagogy concerning 1) how to integrate the curricular materials within an existing program of study and 2) what types of activities and materials should be used. We concentrate on environmental science programs in the US, where structured, instructor-led experiences such as courses or workshops are the norm [32].

Environmental science programs are quite diverse, ranging from water resources to environmental justice to toxicology. The broad topics of expertise, risk, and sociopolitical constraints are pertinent to all of them, but they can manifest quite differently. Many environmental science programs offer stand-alone courses or workshops where different values and policy topics are discussed (e.g., [33,34]). That approach acknowledges that values and policy are areas with their own extensive scholarship, best delivered by faculty with training in these topics. Other programs integrate values and policy topics into existing disciplinary courses [35]. Doing so helps students recognize that values and policy issues permeate science and are not distinct from the technical material and skills they learn. This may be the only available option for programs that have no philosophers or social scientists on their faculty. However, it is widely recognized that scientists may not be confident or comfortable delivering such material or evaluating student performance [36,37].

Recognizing the diversity of environmental science graduate programs and their limited resources, we chose to develop materials that could be customized for use in a wide range of classes, rather than a stand-alone class exclusively covering the material. Given this decision, we needed to develop materials that could be used confidently by faculty with expertise in any discipline and who could not be expected to have formal training in values and policy or evaluating ethical reasoning. We attempted to accomplish this by creating a range of documents that were sufficiently structured to alleviate the need for faculty to have deep expertise in these domains.

Our decisions about content and materials were guided by the strong consensus in the literature that classroom activities promoting social interaction are superior to passive instruction [38,39]. In particular, topics such as those encompassed by our curriculum are productively addressed through activities that clarify values through dialogue [40]. Discussing one another’s views can lead to more clarity about one’s own position, as well as empathy for other positions. However, while such interaction is generally good, care needs to be given to providing the right type and degree of structure to the dialogue. A structured process allows for more efficient and productive exchange of ideas than an unstructured process where students are simply asked for their thoughts or opinions [41]. However, if there is too much structure, students may rush to reach the “right answers” [42]. Therefore, it is recommended that scaffolding be provided, such as discussion prompts that elicit differences among students and require explanation of different points of view [43]. The scaffolding should structure the conversation, but it should also provide for some student freedom, so as to promote shared knowledge construction [44]. Moreover, some scholars have found that student-directed inquiry is more effective than instructor-directed activities [45,46,47].

Ideally, scaffolding for dialogue should promote explanation and argumentation, as opposed to having students announce and defend their points of view [48,49]. Care needs to be taken to encourage students to engage one another, so as to avoid having interactions that consist of an instructor asking questions and students responding [50]. The ideal is to achieve “critical, elaborative discourse” that replaces simple articulation of a position with a cogent justification but that does not devolve into conflictual debate [43,51]. When done well, such dialogue can foster empathy for multiple points of view [52].

#### Values and policy curricular materials

The materials we developed had several features that incorporated principles gleaned from our literature review. First, we developed clear guidance for instructors, including an overview letter, a customizable electronic lecture presentation, a description of the three domains (i.e., expertise, risk, sociopolitical constraints, and non-human impacts), a reading on the use of philosophical dialogue in collaborative science, a set of FAQs, and specific learning objectives. Second, we provided two lesson plans–one for a 6-hour (2-week) implementation and one for a 3-hour implementation–which makes the curriculum adaptable for different situations. The lesson plans detailed activities, assignments, and discussion topics for each hour; we also suggested activities for assessing student learning. (All materials are available from the authors upon request.)

The curriculum involves introductory material and readings to be used in guiding initial class lecture and discussions. Students then engage with case studies that highlight different aspects of the three domains, drawn from readings that focus on environmental science contexts that are related to their own. We provided six examples, but we recognized that they would likely not fit perfectly for many situations. Therefore, we also provided a document to help instructors pick materials that supply locally relevant case studies illustrating the values and policy dimensions we emphasize in our curriculum. One way that students have engaged with these case studies is by role playing them, adopting the different perspectives in the situations as a way of appreciating the different and sometimes incompatible values that are in play. Once they have engaged with these locally relevant case studies, students work in groups to develop “discussion prompts” that serve as scaffolding for the culminating activity, a facilitated classroom discussion among students about aspects of the domains that most engage them. These discussions follow the Toolbox dialogue protocol (http://tdi.msu.edu/), which is designed to support reflexivity and perspective-taking [53]. We supplied a basic list of discussion prompts related to the thematic domains to serve as models for the students when they develop their own prompts, such as the following:

- “Risks identified by people directly affected by a policy should be the primary concern for policy-makers”
- “Policy-makers should always attend to the risks identified by scientific experts”
- “Interdisciplinary environmental scientists must keep their personal values out of their role in the policy process”
- “Interdisciplinary environmental scientists should advocate against policies that limit scientific research they value”
- “Elected officials should set funding priorities in interdisciplinary environmental science.” (We acknowledge that our use of the term *interdisciplinarity* in these prompts departs from the emphasis in the article on *transdisciplinarity*; however, the term “interdisciplinary” here was used to acknowledge that environmental science as typically practiced and as taught in these programs involves multiple disciplinary perspectives. To highlight the fact that it is often transdisciplinary, the prompts also referenced stakeholders, policy makers, and the policy context.)

The curriculum encourages instructors to guide students through a process of developing their own discussion prompts based on issues that arise from their own work or the case studies they examined. The centerpiece of the curriculum is a group discussion structured around the prompts; our materials include guidance for conducting this dialogue and evaluating its quality.

### Hypotheses

Our goal in this study was to evaluate improvement in the sophistication of students’ ethical reasoning about expertise, risk, and sociopolitical constraints. The evaluation was based on a pre-test/post-test, control group quasi-experimental design, in which we scored students’ verbal responses to semi-structured questions. Specifically, we sought to assess whether they could (1) identify the significant features of an environmental problem related to values and policy; (2) understand how these features should figure into appropriate stakeholder engagement in research and decision making; and (3) consider the social and ethical tradeoffs among different scientific, policy, and management decisions related to expertise, risk, and sociopolitical constraints.

Our three hypotheses were:

- Students in experimental classes would not differ from students in control classes at the pre-test;
- Students in the experimental classes would show significant improvement in ethical reasoning from pre-course to post-course; and
- Students in the experimental classes would score more highly on reasoning at the post-test than students in the control classes.

## Methods

### Quasi-experimental design

This research was approved by the Institutional Review Boards of Michigan State University, the University of Idaho, and Oregon State University. Informed consent was obtained verbally. Data were generated through interviews in which students were presented with an environmental problem and asked to explain their views on specific questions related to expertise, risk, and sociopolitical constraints at each phase of the research process. Structuring the interview through the analysis of a scenario accomplished two goals: it avoided abstract, unfamiliar, and potentially frustrating discussion of ethical theory, and it allowed us to evaluate student reasoning in a practical context such as they might face upon completion of their educational programs. A drought management scenario was used in pre-course interviews, while an invasive species management scenario was used in the post-course interviews (see S1 File); different pre- and post-course scenarios were selected to discourage students during the post-test from feeling committed to answers they had provided in the pre-course interviews. (This approach is similar to the one used by Remington-Doucette, Hiller Connell, Armstrong, and Musgrove [54], which used different pre-test and post-test case studies and a rubric to evaluate student responses.) The description of each scenario stated that stakeholders contested both the scope of the problem and the preferred response.

We conducted semi-structured interviews to assess whether students who participated in the curriculum developed more sophisticated ethical reasoning about the three domains than students who did not participate. For the purposes of this study, we take *ethical reasoning* to involve the articulation of widely acceptable reasons for how to attribute expertise, evaluate risk, or navigate sociopolitical constraints. Given this, *more sophisticated ethical reasoning* entails a greater awareness of considerations relevant to expertise, risk, and sociopolitical constraints, along with an enhanced ability to provide reasons for positions taken on these issues. For instance, students faced with the challenge of evaluating risk should appreciate that those affected should play key roles in weighing different risks, while those faced with the challenge of attributing expertise should appreciate that experts’ authority will depend on a community’s history and trust [55,56].

During the control year, we interviewed 15 graduate students in five courses from one medium and one large public university in the United States. In the experimental year, we interviewed 27 students in the same courses at the same institutions. We conducted two interviews with each student–one at the beginning and one at the end of the semester–in both the control and experimental years. In the experimental year students were exposed to our pedagogical materials between the pre- and post-course interviews; students in the control year were not exposed to these materials. The post-course interviews in the experimental year were conducted approximately one to two months after the implementation of our materials. By comparing the extent of growth in ethical reasoning between the pre-course and post-course interviews between the control and experimental years, we are able to determine the effectiveness of our pedagogical materials.

Students spent the first 15 minutes of the interviews reading the scenarios and writing down which actors they felt should be involved in addressing the problem at the four phases of the research process: (1) conceptualizing the problem, (2) collecting information, (3) analyzing/interpreting information, and (4) reaching a decision. They were asked to draw a visual diagram of the phases, showing the stakeholders involved, which served as a conversation aid throughout the interview. In addition to identifying the actors who should be involved at each phase of the process, students were asked to indicate what information (and from whom) would be necessary to make informed and appropriate decisions at each phase, and how the various actors should make decisions based on this information. Interview questions elicited explanations of the reasoning underlying their answers.

Three questions (capturing specific issues of expertise, sociopolitical constraints, and risk) were asked in relation to each of the four phases of the research, resulting in 12 questions that focused on the three domains of interest in this paper. One of the questions under sociopolitical constraints proved confusing to students and was ultimately dropped from analysis, so the analysis in this paper is based on 11 questions (Table 1). These questions were derived from recent literature on the intersection of science, values, and policy; basing questions on well-theorized ethical issues introduced an account of best practices against which student reasoning could be compared (e.g., [57,58,59]). For example, when considering “expertise” during the decision-making phase, students were asked whether researchers should advocate for particular responses, with follow-up questions that encouraged students to distinguish contexts where advocacy would be appropriate and contexts where advocacy would be problematic. This phase of the interview typically spanned 30–40 minutes.

**Table 1:** Interview questions for expertise, risk, and sociopolitical constraints^+^.

Domain	Phase of research			
	Conceptualizing the problem	Collecting information	Analyzing and interpreting information	Decision-making
**Expertise**	What inputs should different stakeholders contribute? Who are the experts and what makes them experts? (1a)	If the public believes information is relevant, but researchers disagree, should that information be collected? (1b)	If two researchers use different methods that lead to different conclusions, how should this be resolved? What is the role of the public? (1c)	Should the researchers make specific policy recommendations? (1d)
**Risk**	What harms should be considered when deciding what aspects of the problem to study, and does it matter whom or what these harms might affect? (2a)	How much information should be collected? If the public challenges how researchers measure risks, how should researchers respond? (2b)	How should researchers consider environmental impacts or possible solutions that the public considers to violate basic rights? (2c)	What should researchers consider when deciding how certain they should be in their findings before bringing them to decision-making? (2d)
**Sociopolitical constraints**	Considering that findings could have implications for policy, should researchers explore research questions that are unpopular or unlikely to receive political support? (3a)	If scientists think information would support a politically unpopular or divisive outcome, should they collect the data?^^^	How should researchers choose between methods that are rigorous but esoteric and methods that are less rigorous but more resonant with stakeholders? (3c)	What stakeholders should decide on the response to address the problem? How should researchers participate if their findings support an unpopular response? (3d)

^+^Notations in the table (e.g., 1a, 2a, etc.) will be used in Tables 4, 5, 6 and 7 to illustrate the questions and their associated mean scores.

^^^This question was removed from the analysis because students at both institutions responded in disparate ways which we were unable to score.

### The courses

The five courses in which we implemented our materials represent a diversity of topics and disciplines within environmental science (Table 2). As is typical in graduate-level, transdisciplinary environmental science programs, each course represents a unique interdisciplinary nexus that fits the distinctive goals of its program. For example, “Community-Based Natural Resource Management in Developing Countries” integrates natural resource management, global development studies, and economics to provide students with the essential perspectives to broadly understand topics such as land tenure and the role of natural resources in rural livelihood systems. In another example, “Interdisciplinary Methods in Water Resources” integrates water resources, research methods, and ethics to provide students with the skills they need as they enter professions related to water management. Despite the differences between the courses, all share a commitment to both topical diversity and interdisciplinary integration.

**Table 2:** Information about courses in which the curriculum was implemented.

Course name	Instruction and instructor background(s)	Credits	Interdisciplin-ary Nexus	Curriculum implementation	Program Context	Typical Students	Control year (n)	Implement-ation year (n)
**Ecological Food and Farming Systems Seminar: Interdisciplinary Approaches to a Changing World**	Team	1	Sustainable food systems and agriculture	Over a 3-week period in the beginning of term	An optional course in a graduate degree program devoted to community sustainability	Master of Science (M.S.) and Ph.D. graduate students in community sustainability and related programs in agriculture and natural resources	2	3
	Instructor 1: Discipline: Terrestrial ecologist (urban and agricultural); taught course 3 times prior to baseline year Instructor 2: Discipline: Food science and sustainable agriculture; taught course 3 times prior to baseline year							
**Community-Based Natural Resource Management in Developing Countries**	Individual	3	Natural resource management, global development, and economics	Over a 4-week period late in the term	An optional course in a graduate degree program devoted to community sustainability	M.S. and Ph.D. graduate students in community sustainability and related programs in agriculture and natural resources	1	7
	Instructor: Discipline: Resource economist; taught course 1st time during baseline year							
**Introduction to Environmental Science and Policy**	Team	3	Policy and environmental science	Over a 2-week period in the middle of term	A required course for first year participants in a graduate program focusing on the science, engineering, and policy aspects of environmental issues	M.S and Ph.D. graduate students pursuing a graduate option in environmental science and policy	3	4
	Instructor 1: Discipline: Complex systems modeler; taught course 3 times prior to baseline year. Instructor 2: Discipline: Environmental engineer; taught course 1st time during baseline year							
**Interdisciplinary Methods in Water Resources**	Team	3	Water resources, research methods, and ethics	Over a 2-week period in the middle of term	A required course in an interdisciplinary water resources management graduate program	Ph.D., M.S., and Juris Doctor (J.D.) students enrolled in the water resources management program, as well as other environmental science graduate students	3	5
	Instructor 1: Discipline: Water law; taught course 6 times prior to baseline year. Instructor 2: Discipline: Hydrology; taught course 5 times prior to baseline year							
**Advanced Field Ecology Course Design**	Individual	5	Field-based education, science communication, and ecology	Over a 6-hour single period	A required course in a graduate program for K-12 science teachers that focuses on environmental education and science communication	M.S. students who are typically employed as science teachers in elementary, middle, and high schools	6	8
	Instructor: Discipline: Environmental social science/education; taught course 1 time prior to baseline year							

Our curriculum affords instructors discretion in how it is implemented in their courses. This latitude was evident as the instructors across the five courses utilized the materials in somewhat different ways (Table 2). For example, the six-hour curriculum was implemented at different points in the term by different instructors. Additionally, the curriculum was dispersed across two, three, or four weeks and, in one course, was even condensed into a single long session. There were also differences in the ways curricular materials were used. The opening, customizable lecture presentation created to introduce the domains was used by some but not all of the instructors, and different decisions were made about which case study examples to use in developing the domains in each particular course context. Yet, despite the differences in implementation, all courses included the core aspects of our curriculum: consideration of the domains emphasized in the curriculum, student-led development of discussion prompts related to the curriculum’s domains, and student dialogue about the issues articulated in the discussion prompts.

### Analysis

To determine whether students developed more sophisticated ethical reasoning about values and policy as a result of participation in our curriculum, we developed a rubric to score the responses to each structured interview question, using a scale from zero (no awareness of ethical issues) to four (sophisticated reasoning about issues and contexts). Using this rubric, statements suggesting a lack of awareness of relevant issues or vague distinctions (e.g., saying that input from the public should be excluded in a phase of research, without articulating a reason for such exclusion) were scored lower than sophisticated distinctions (e.g., articulating a reason to prioritize public perspectives when members of the public are uniquely vulnerable to an environmental problem). We subsequently explored the qualitative data in depth to understand the nature of the changes occurring in students’ reasoning [60].

Students whose reasoning aligned with expert literature (e.g., [61,62]) were taken to demonstrate advanced ethical reasoning skills (Table 3). Basic reasoning was associated with cognitive skills such as distinguishing and recognizing issues, whereas more advanced reasoning made use of recognized distinctions by giving reasons for a course of action or weighing tradeoffs between different actions, in addition to distinguishing and recognizing [15]. In effect, students who had command of the three domains were able to recognize values and policy questions (e.g., “who is an expert in what?” or “who determines the acceptability of different risks?”), anticipate various answers to these questions, and understand that policy decisions among competing values should be decided through democratic deliberation [16,62,63]. “Growth” in sophistication of reasoning was evident when students demonstrated more advanced reasoning in the post-course interview than they had demonstrated in the pre-course interview. For example, a student, in his or her pre-course interview, might have appreciated that different disciplines offer different, valid methods for dealing with a particular environmental problem but not have considered the different implications of these disciplinary perspectives for policy outcomes. If the student later offered a more sophisticated discussion of the merits of different methods, or (better yet) discussed tradeoffs between these methods, s/he would receive a higher score for the post-course interview.

**Table 3:** Differences between basic and advanced reasoning in participant interviews.

Domain	Basic reasoning	Advanced reasoning
Expertise	• Distinguished between input from stakeholders and scientists • Recognized that scientists are accountable to stakeholders • Recognized that values inform the selection of research methods	• Provided reasons for limiting the scope of scientific expertise • Provided reasons for incorporating the knowledge of scientists or local actors • Weighed tradeoffs at stake when deciding whether to advocate for policy as a scientist
Risk	• Distinguished between factual and evaluative questions • Proposed stakeholder involvement to resolve differences in the evaluation of risks	• Provided reasons stakeholders should take the lead in the evaluation of risks • Anticipated criteria relevant for prioritizing particular values (e.g., vulnerability) • Weighed tradeoffs at stake in deciding whether to collect additional data
Sociopolitical constraints	• Recognized constraints on scientific investigation (e.g., limited time/resources) • Recognized that scientists confront decisions about how to interface with policymakers and the public	• Weighed tradeoffs at stake in selecting familiar or unfamiliar research or analysis methods • Weighed tradeoffs at stake in suggesting alternative interventions

We developed the scoring rubric through several rounds of application, discussion, and refinement within the research team. Two scorers used the final rubric to evaluate all interviews independently, and the inter-rater reliability across all scores was strong (κ = .84; p < .0005; [64]). Discrepancies between scores were reconciled by discussing alternative interpretations of student positions and identifying which of these interpretations was most warranted.

A small number of responses to questions were unscorable. Most of these unscorable responses occurred when students did not understand the thrust of the question being asked. Interviewers tended to ask one or two follow-up questions to steer them in the “proper” direction (i.e., toward the content covered in our curriculum), but if participants were not responsive to this, interviewers would move on so as to avoid putting pressure on participants or making them think they were wrong. Across all responses in the interviews, only 25 out of 924 were unscorable.

To evaluate the magnitude of change in response quality, we used non-parametric tests with an alpha level of .10. We used Mann-Whitney *U* tests to compare the pre-test scores from the control and implementation years and to compare the post-test scores from control and implementation years. We used Wilcoxon signed-rank tests to compare the pre- and post-test scores for each year. Effect size statistics (*r*) are also reported. Following the quantitative results, we qualitatively explored responses from the pre-test to identify typical tendencies in initial reasoning. We then explored responses from students who exhibited meaningful improvements in reasoning to understand the nature of improvements. We use excerpts from interviews to illustrate these patterns.

## Results

### Changes in students’ ethical reasoning: Quantitative results

Overall, students’ level of reasoning was low at the pre-test for both control and implementation years (Table 4), with mean scores ranging from 1.2 to 2.5 on the scale of zero to four. Consistent with expectations, most of the pre-test scores did not differ between the control and implementation year. For one item (expertise, analysis and interpretation phase), control students scored higher than implementation year students. This difference was statistically significant (p ≤ .10) with a medium effect size [65]. Overall, however, the groups seem fairly well matched.

**Table 4:** Comparison of control and implementation groups' pretest scores.

Phase	Domain^a^	Year^b^	n	Mean	*U*	z	*P*	*r*
**Conceptualizing**	**Expertise (1a)**	**C**	13	2.46	138.50	-1.15	.25	-.24
		**I**	27	2.19				
	**Risk (2a)**	**C**	15	2.27	156.50	-1.40	.17	-.22
		**I**	27	1.96				
	**Sociopolitical constraints (3a)**	**C**	14	1.43	176.00	-.18	.86	-1.14
		**I**	26	1.42				
**Data Collection**	**Expertise (1b)**	**C**	14	2.00	178.00	-.32	.75	-2.05
		**I**	27	1.96				
	**Risk (2b)**	**C**	15	1.80	176.50	-.74	.46	-.11
		**I**	27	2.00				
**Analysis and Interpretation**	**Expertise (1c)**	**C**	14	2.36	119.00	-2.04	.04	-.32
		**I**	27	1.56				
	**Risk (2c)**	**C**	12	1.75	105.00	-1.40	.16	-.23
		**I**	24	2.46				
	**Sociopolitical constraints (3c)**	**C**	14	1.64	155.50	-1.12	.26	-.17
		**I**	27	2.04				
**Decision-making**	**Expertise (1d)**	**C**	15	1.20	183.00	-.56	.58	-.09
		**I**	27	1.48				
	**Risk (2d)**	**C**	14	1.57	198.00	-.21	.83	-.03
		**I**	25	1.44				
	**Sociopolitical constraints (3d)**	**C**	14	1.43	183.00	-.18	.86	-.03
		**I**	27	1.48				

^a.^ The notations (1a, 2b, etc.) refer to interview questions found in Table 1.

^b.^ “Control” has been abbreviated as “C” and “implementation” as “I”.

Given the absence of treatment in the control year, we did not expect control students’ pre-test and post-test scores to differ. Among the 11 scores, two showed a statistically significant change, with medium effect sizes: scores increased for expertise (in the conceptualizing phase) and for risk in the data collection phase (Table 5). Of 153 pairs of responses across all questions for all study participants, 92 showed no change from pre-test to post-test, 40 increased by at least one point, and 21 decreased by at least one point. Collectively, the lack of consistent change generally supports the conclusion that these classes were not imparting instruction related to the three themes prior to adoption of our curriculum.

**Table 5:** Control year–comparison between pre- and post-implementation scores^a^.

Phase	Domain^b^	Pre or post	Mean	n	*z*	*p*	*r*
**Conceptualizing**	**Expertise (1a)**	**Pre**	2.46	13	-1.63^c^	.10	-.32
		**Post**	2.77	13			
	**Risk (2a)**	**Pre**	2.27	15	-.28^c^	.78	-.05
		**Post**	2.33	15			
	**Sociopolitical constraints (3a)**	**Pre**	1.43	14	-1.30^d^	.20	-.25
		**Post**	1.07	14			
**Data Collection**	**Expertise (1b)**	**Pre**	2.00	14	-1.00^c^	.32	-.19
		**Post**	2.21	14			
	**Risk (2b**	**Pre**	1.80	15	-2.64^c^	.01	-.48
		**Post**	2.47	15			
**Analysis and Interpretation**	**Expertise (1c)**	**Pre**	2.31	13	.00^e^	1.00	0.00
		**Post**	2.31	13			
	**Risk (2c)**	**Pre**	1.75	12	-.59^d^	.56	-.12
		**Post**	1.50	12			
	**Sociopolitical constraints (3c)**	**Pre**	1.64	14	-1.38^c^	.17	-.26
		**Post**	2.14	14			
**Decision-making**	**Expertise (1d)**	**Pre**	1.20	15	-1.06^c^	.29	-.19
		**Post**	1.53	15			
	**Risk (2d) (2d)**	**Pre**	1.57	14	-.38^c^	.71	-.07
		**Post**	1.64	14			
	**Sociopolitical constraints (3d)**	**Pre**	1.43	14	-1.41^c^	.16	-.27
		**Post**	1.71	14			

^a.^ Wilcoxon Signed Ranks Test.

^b.^ The notations (1a, 2b, etc.) refer to interview questions found in Table 1.

^c.^ Based on negative ranks.

^d.^ Based on positive ranks.

^e.^ The sum of the negative ranks equals the sum of the positive ranks.

Comparing the control and implementation groups’ post-test scores identified four measures with statistically significant differences, with students in the implementation year scoring higher than in the control year (Table 6). Two of these were for the analysis and interpretation phase (risk and sociopolitical constraints), while one was in the conceptualization phase (sociopolitical constraints) and the other was in the data collection phase (expertise). All these changes were statistically significant (*p* ≤.10); effect sizes were medium, except for the domain of sociopolitical constraints in the analysis and interpretation phase, which was small [65].

**Table 6:** Comparison of control and implementation groups' post-test scores.

Phase	Domain^a^	Year^b^	n	Mean	*U*	*z*	*P*	*r*
**Conceptualizing**	**Expertise (1a)**	**C**	15	2.80	189.00	-.48	.63	-.07
		**I**	27	2.85				
	**Risk (2a)**	**C**	15	2.33	164.50	-1.10	.27	-.17
		**I**	27	2.63				
	**Sociopolitical constraints (3a)**	**C**	15	1.27	125.00	-1.90	.06	-.30
		**I**	25	1.92				
**Data Collection**	**Expertise (1b)**	**C**	15	2.13	121.50	-2.21	.03	-.35
		**I**	26	2.69				
	**Risk (2b)**	**C**	15	2.47	179.00	-.47	.64	-.07
		**I**	26	2.58				
**Analysis and Interpretation**	**Expertise (1c)**	**C**	14	2.43	148.50	-1.18	.24	-.18
		**I**	27	2.93				
	**Risk (2c)**	**C**	15	1.60	123.00	-2.11	.04	-.33
		**I**	26	2.46				
	**Sociopolitical constraints (3c)**	**C**	15	2.20	138.00	-1.64	.10	-.26
		**I**	26	2.81				
**Decision-making**	**Expertise (1d)**	**C**	15	1.53	189.00	-.17	.87	-.03
		**I**	26	1.62				
	**Risk (2d)**	**C**	15	1.73	192.50	-.27	.78	-.04
		**I**	27	1.59				
	**Sociopolitical constraints (3d)**	**C**	15	1.73	95.00	-.21	.83	-.03
		**I**	27	1.67				

^a.^ The notations (1a, 2b, etc.) refer to interview questions found in Table 1.

^b.^ “Control” has been abbreviated as “C” and “implementation” as “I”.

Finally, among students receiving the curriculum, there were seven cases where measures improved significantly (*p* ≤ .10) from pre-test to post-test (Table 7). Three of the four measures from the domain of expertise showed improvement; two of the measures related to sociopolitical constraints and two of the measures related to risk improved. All but two of these changes had medium effect sizes: the domain of sociopolitical constraints in the conceptualization phase had a small effect size and the expertise domain in the analysis and interpretation phase had a large effect size [65]. Across all 284 pairs of responses, 112 did not change, 134 increased by at least one point, and 38 decreased by at least one point.

**Table 7:** Implementation year–comparison between pre- and post-implementation scores^a^.

Phase	Domain^b^	Pre or post	Mean	n	*z*^*c*^	*P*	*r*
**Conceptualizing**	**Expertise (1a)**	**Pre**	2.19	27	-3.14	< .01	-.43
		**Post**	2.85	27			
	**Risk (2a)**	**Pre**	1.96	27	-3.22	< .01	-.44
		**Post**	2.63	27			
	**Sociopolitical constraints (3a)**	**Pre**	1.50	24	-1.70	.09	-.25
		**Post**	1.96	24			
**Data Collection**	**Expertise (1b)**	**Pre**	1.92	26	-2.62	< .01	-.36
		**Post**	2.69	26			
	**Risk (2b)**	**Pre**	1.96	26	-2.57	.01	-.36
		**Post**	2.58	26			
**Analysis and Interpretation**	**Expertise (1c)**	**Pre**	1.56	27	-3.84	< .01	-.52
		**Post**	2.93	27			
	**Risk (2c)**	**Pre**	2.43	23	-.26	.80	-.04
		**Post**	2.48	23			
	**Sociopolitical constraints (3c)**	**Pre**	2.04	26	-3.04	< .01	-.42
		**Post**	2.81	26			
**Decision-making**	**Expertise (1d)**	**Pre**	1.42	26	-.84	.40	-.12
		**Post**	1.62	26			
	**Risk (2d)**	**Pre**	1.44	25	-.85	.39	-.12
		**Post**	1.64	25			
	**Sociopolitical constraints (3d)**	**Pre**	1.48	27	-.85	.40	-.12
		**Post**	1.67	27			

^a.^ Wilcoxon Signed Ranks Test.

^b.^ The notations (1a, 2b, etc.) refer to interview questions found in Table 1.

^c.^ Based on negative ranks.

### Qualitative results

While the quantitative results above provide an indication of the extent and magnitude of change, they do not illuminate the nature of the changes in students’ reasoning. To understand how awareness of the domains and reasoning about them expanded or deepened, we turned to the interview transcripts. Below we use excerpts to illustrate common tendencies we observed in the data. Examining these transcripts closely reveals certain patterns of change in student reasoning in relation to questions for each of the research phases. Overall, these changes can be characterized as shifts from uncritical adherence to the practices of technical experts and responses to more well-justified and epistemically viable inclusion of relevant public actors who play unique–and important–roles at each phase of the research process.

#### Problem conceptualization phase of research

In the problem conceptualization phase, improvements tended to reflect a broadening of student recognition of the roles for the public. Pre-course responses were often characterized by general exclusion of the public from this research phase. For example, one student felt that scientific researchers should purposefully exclude the public’s voice in defining or conceptualizing the socio-environmental problem because “it is the right thing to do”; this student felt that the end product of this stage should reflect only scientific expertise, “even if it’s not what the public wants.”

In post-course interviews, students were more aware of the need for meaningful engagement between scientists and the public when formulating research. Specifically, this engagement was characterized by the inclusion of the public’s local expertise and knowledge and their perceptions of what risks and harms are acceptable, especially among marginalized or underrepresented groups. For example, a student argued that local residents–as experts about their unique local circumstances–should provide input about how the problem presented in the scenario is “affecting them” and what the public sees as a desired future condition. In a response that reflects meaningful consideration of marginalized peoples, another student felt that it was important to “canvass” (i.e., systematically compile input from) people who are “underrepresented in politics” to ensure they are not harmed in unanticipated ways.

In an example of how one student’s responses meaningfully changed from pre-test to post-test, the student was given a low score (1) in the pre-interview because the student did not provide an adequate rationale or justification for the inclusion of the public’s perspective in this phase. However, in the post-interview, the student received a higher score (3) because of an expressed recognition that the public “may have something important to say” due to their local expertise about the problem. This student also asserted that formal mechanisms should be put in place to allow members of the public to have their voices heard, especially “quiet” members of the public.

Positive change between pre-test and post-test scores during the implementation year for individual students was evident in the conceptualizing the problem phase of the research process. In this phase, 11 of 27 students increased by at least one level in the domain of expertise and no students scored lower at the post-test; 14 increased at least one level on risk and one student scored lower in this phase; 10 increased in sociopolitical constraints, while five received lower scores in this phase.

#### Data collection phase of research

In the data collection phase, low scores were often due to a failure to recognize any role for the public in generating information relevant to addressing environmental issues. For example, one student stated in a pre-interview that the public is “not in the position to question how scientists are collecting” data. Another tendency was for students to provide inadequate justification for including public input (e.g., such inclusion should only occur to placate members of the public in a process that should “rightly” be driven by scientists). For example, one student received a low score in the pre-interview based on his response that the inclusion of public input was only important because allowing “people to feel like their voices are heard” facilitates public buy-in of the process.

Students who received higher scores envisioned a more meaningful engagement between scientists and the public in data generation. This tended to take one of two forms: engagement in which scientists must provide publicly understandable scientific justification for their decisions to include particular types of data, or the public taking a lead role in identifying information pertinent to publicly perceived risks or harms. In an example of the first, one student in a post-course interview felt that scientists should “demonstrate” the importance of the information they are collecting to reflect public interests and “try to explain it [to the public] on a basic enough level that it makes sense”; this response received a relatively high score because it recognized the social obligation of scientists to communicate effectively with the public and acknowledge public concerns. In an example arguing for public ability to identify information related to perceived risks or harms, another student said that members of the public should be involved at this stage because they are attuned to harms related to the socio-environmental problem in the scenario. That is, the public is “part of it” and understands its “cost to the local community”; this response exemplifies the public taking a lead role in identifying information pertinent to perceived risks or harms.

Positive change between pre-test and post-test scores during the implementation year for individual students was evident in the data collection phase of the research process. Fifteen students improved in the domain of expertise, while five students regressed; 14 improved their risk scores, while four regressed. (Interview responses related to the domain of socio-political constraints in the data collection phase of the research process were unscorable because of the disparate ways students answered and therefore were not included.)

#### Analysis and interpretation phase of research

In the analysis and interpretation phase of the research process, responses with low scores (0 or 1) were typically characterized by an abiding commitment to technical scientific approaches without taking the social context of science into account. For example, one student responded that if two different methods lead to different results, then “one must be wrong because one has to be better than the other,” and another stated that “it’s hard to imagine two different methods getting different answers.” A more sophisticated response to the interview questions asked during the analysis and interpretation phase would have argued that the research team should meaningfully consider the public’s weighing of risks and trade-offs associated with the different approaches. In an example that reflects this more advanced reasoning, one participant stated that there are “so many different data sets and values and perspectives coming to the table and the point isn’t to make them all make sense.” For this participant, the point was to understand the “trade-offs” of relying on each of these perspectives in the context of mitigating the socio-environmental problem for the public in the least harmful way possible.

In discussing this phase of research, 20 students from the implementation year showed improvement in the domain of expertise, while two regressed; 14 improved and two regressed in sociopolitical constraints in this phase. For the domain of risk, there were mixed changes at the individual level as six improved, while five regressed.

#### Decision-making phase of research

In the decision-making phase, students were given low scores (0 to 1) when they felt that there were no instances in which a scientist should advocate for policy outcomes or did not articulate contextual factors that may appropriately lead to advocacy. For example, a student who received a low score for this phase did “not feel like scientists should [advocate],” but rather that scientists should uncritically accept “whatever the stakeholders choose” in terms of a response to the problem. Another student argued that scientists should simply share “what they found,” because “it’s really the policy makers that have the last say.” Low scores were also given to students who did not consider public values in contexts where results were uncertain, but simply responded in ways such as this: “you can’t account for everything, and so you are probably going to have a degree of error”; “you can’t consider everything” because there is “uncertainty in everything”.

Although significant positive changes between pre-test and post-test for the experimental year were seen in the majority of domains in the first three phases of the research process, these changes were not evident in the last stage, decision-making. None of the measures associated with the decision-making phase of research showed statistically significant change (Table 7). This likely reflected the mixed patterns of changes between pre-test and post-test scores among implementation year students. Specifically, nine students improved but five regressed in the domain of expertise; for risk in this phase, 10 improved, but six regressed; and finally for sociopolitical constraints in this phase, nine improved while four regressed.

## Discussion

### Summary of findings

Our pre-test findings confirm that the domains addressed by our curriculum–expertise, risk, and sociopolitical constraints–are not topics in which new environmental science graduate students are well versed. On the 5-point scales we used, where zero was the lowest possible score and four was the highest, the mean pre-test values were mostly less than 2.0. It was clear from the interviews with students that most of them had not given much thought to these issues, as they frequently had difficulty articulating reasons for their views. For some items, students had largely consistent views, but these lacked refined awareness of the realities of transdisciplinary research. For example, nearly all of them believed that the public should have a role in conceptualizing the problem to be studied. However, when pushed for further elaboration on this, they tended to argue positions such as that “everyone” with a stake should be able to influence the process. Responses like this neglect the complex realities inherent in situations where different stakeholders hold different values, are differentially impacted, and have different views on what should be done. This lack of awareness and nuance reinforces the need for curricula that help students reflect more deeply on these ethical issues.

As we noted above, the curriculum was implemented in varied ways across the five classes, but all had students develop discussion prompts relevant to their unique circumstances and engage in a group dialogue around the prompts. Some classes incorporated locally relevant readings and case studies, while others relied solely on the readings and materials we supplied. Despite these differences, inspection of the individual changes on each of the 11 scored elements showed similar amounts of change across all classes. Given this finding, as well as the absence of statistically significant differences between control and treatment groups at the pre-test, the significant improvement in reasoning among treatment students, and post-test differences between control and treatment groups, we conclude that the most plausible interpretation is that the materials we developed were effective in imparting knowledge and improving reasoning about values and policy in transdisciplinary environmental research.

Delving into the 11 scored elements, improvements were evident for all three domains in three of the four phases of research. In the domain of expertise, after the curriculum, students exhibited expanded awareness of the need to include and prioritize public input as a legitimate form of expertise. There was greater recognition that the public should lead in deciding what and how to study when it comes to problem-related matters, and that scientists are often more rightly assigned expertise related to epistemic matters. Moreover, students increased their awareness and reasoning about how public values and knowledge may rightly affect which methods are used to generate information pertinent to the problem, and that science is not always a matter of one form of knowledge being “right” and others being “wrong.” Instead, students became more able to articulate that such conditions do not simply indicate “bad” science, but that different methods may generate valid and useful findings; they also improved their ability to articulate the need to communicate such issues transparently to stakeholders.

In the domain of risk, which relates to the types of harms associated with environmental problems and the levels of uncertainty about them, after exposure to the curriculum, students demonstrated enhanced awareness that all stakeholders should be enabled to provide input about the types of harms to be assessed and provided better arguments about why the research process should prioritize the study of risks according to public concerns. When discussing risk issues in the context of the data collection phase, they showed increased ability to discuss the tradeoffs associated with different levels of uncertainty and the desirability of collecting more data or data on issues of concern to the public.

In the domain of sociopolitical constraints, students demonstrated greater ability after the curriculum to articulate tradeoffs associated with research that have implications for actions or policies that are politically or publicly controversial. They also showed more recognition that scientists acting in the analysis and interpretation phase need to consider the public comprehensibility of policy-relevant science, and not simply select the most sophisticated forms of analysis available. That is, students moved from a position of advocating for the “most accurate” analysis to a recognition that methodological and analytical choices may affect the public’s reaction to science, and therefore to subsequent policy decisions.

It is interesting to note that in the decision-making phase there was no statistically significant change in any of the three values and policy domains. Although we cannot be certain, we suspect that the principal reason for this is the lack of attention to decision-making in the implementation of the curriculum. The questions in the decision-making phase asked about the level of certainty scientists need before presenting their findings in a policy context, whether scientists should make policy recommendations, how scientists should be involved if their findings could be construed as supporting unpopular actions, and who should ultimately make policy decisions. These concerns were not present in most of the sets of discussion prompts produced by students in the five courses, indicating that the elements in this phase were not emphasized in the implementation of the curriculum across the five courses. Given the importance of these issues, a focus should be placed on increasing attention to them when using our curriculum in the future.

### Recommendations for transdisciplinary environmental science programs

Many authors have noted that issues of values and policy are often outside the expertise of faculty in environmental science programs [7,8]. Therefore, clear materials are needed to help instructors guide discussions of values and policy among their students. Our curricular materials were informed by relevant literature, and our experience testing them in five classes provides support for certain recommendations. First, materials must either be relevant to specific circumstances of each program or customizable so that they can be made relevant [66,34]. In our case, customizability was enhanced by providing examples, FAQs, and specific guidance for instructors to develop their own approaches and activities. Second, case studies and examples need to be carefully designed to promote discussion and reflection on key issues [13]. We provided readings that could be assigned as homework, and we encouraged instructors to identify their own readings, which most of them did. Third, modules should make time for meaningful group discussion of the themes of the curriculum, since group discussions increase student satisfaction with this type of material and improve student performance [67,68]. The cornerstone of the curriculum was the final group dialogue, and most implementations also had group discussions about materials prior to the ultimate group dialogue. Fourth, if group discussion is built into the curriculum, students need the opportunity to contribute to the structure of that discussion. Based on recommendations from the literature for scaffolding, we created model prompts that could promote discussion in which students identify areas of commonality or valid new perspectives [40]. In most cases, faculty had students develop their own discussion prompts, which likely enhanced the utility of the curriculum for each specific context.

### Limitations and future research

In developing and evaluating our curriculum for transdisciplinary environmental science graduate programs, we made a number of decisions to render the project manageable that also represent limitations on the study. First, there was the decision made about the number and variety of courses in which we implemented the curriculum. Although we selected courses with a range of topics and disciplinary emphases, our study was limited to five classes and a relatively small sample of students. Further, by building flexibility into the curriculum (e.g., a 6-hour curriculum as well as a 3-hour curriculum), we made it possible for instructors to implement it in ways that best aligned with their course constraints; however, this meant that the curriculum was interpreted and imparted differently by each of the instructors who partnered with us. In this case, a strength of the curriculum became a limitation of the experimental evaluation. In addition, due to small sample sizes in each class, we could not include the instructor’s academic background, experience, and pedagogical philosophies as variables in our analysis, all of which may have affected curriculum outcomes. Future research should investigate the curriculum’s effectiveness with a larger and even more diverse sample, while controlling the way in which it is implemented and also considering the potential influence of the diverse backgrounds of instructors in transdisciplinary environmental science graduate programs.

Second, we had to meet the challenge of developing an approach to identifying improvements in ethical reasoning about values and policy. Our materials dealt with quite specific topics, so existing rubrics like those from Association of American Colleges and Universities [69] were too broad for our purposes. Moreover, like Remington-Doucette et al., we used different scenarios for the pre-test and post-test, and this made it slightly more challenging to identify improvements in reasoning [49]. Therefore, we used expert literature on values and policy in environmental research and decision-making to develop a more sensitive, topic-specific rubric for evaluating student reasoning.

Third, we developed specific topics in the curriculum in order to ensure that it supplied enough thematic content to be useful to non-experts. Additional topics could have been included, such as impact on future generations and environmental justice, but given the desire to make it possible to implement the curriculum within a 6-hour timeframe, we decided to limit the topics to a manageable number. The curriculum is designed, however, to support introduction of new topics by knowledgeable instructors who wish to focus their classes on different values and policy themes, either as additional points of emphasis or in place of existing themes.

## Conclusion

In this article, we have detailed several encouraging findings from a formal evaluation of a dialogue-based curriculum designed to enhance awareness of and reasoning about values and policy in interdisciplinary environmental science. Our work in developing and evaluating this curriculum was driven by several motivations. First, there is a need for educational materials related to the ethical dimensions of scientific practice and its social consequences, and we have sought to supply a set of resources that addresses this need [34,14,70,71] . In the interest of providing material that is specific enough to be of interest to graduate students across the broad suite of environmental sciences, we focused our curricular development effort on three topics that relate to values and policy: expertise, risk, and sociopolitical constraints. Second, heeding the call reported by Hall et al. [32] for self-contained, modular curricular elements, we designed the curriculum to be deployed in 6-hour and in 3-hour units that could be modified to fit many different environmental science contexts and course syllabi [34]. Third, we wanted to follow educational best practices and integrate case studies and interactive dialogue into the curriculum. Finally, we were committed to conducting a formal evaluation of the curriculum, implemented in a range of graduate courses, to determine if there was evidence of effectiveness. Although our results are not uniformly strong, there is good reason to believe that students who participate in this curriculum improve their awareness of and reasoning about expertise, risk, and sociopolitical constraints in ways that enhance their training.

## Supporting information

S1 File Text used during interviews. (DOCX)
