# Supplementary material for: Evaluating a dialogue-based approach to teaching about values and policy in graduate transdisciplinary environmental science programs
Source: PLoS One. 2018 Sep 4;13(9):e0202948. doi: 10.1371/journal.pone.0202948 (PMC6122781; doi:10.1371/journal.pone.0202948)
Supplement: S1 File — (DOCX) [file pone.0202948.s001.docx]

**S1 Scenarios Used during Interviews**

**Case study I: Scientific evaluation and the ethical dimensions of a water policy decision in San Antonio**

The city of San Antonio, Texas anticipates water shortages beginning in the next twenty years. While experts, policy makers, and the general public agree that the city should take steps to minimize the impact of these water shortages, there is no consensus about what steps should be taken, and all believe that more information is needed. Some stakeholders believe that the city should introduce new regulations that discourage the use of freshwater by some industries in the region. Others believe that the city should invest in the design and construction of new water infrastructure that will extend the life of existing freshwater sources and/or tap into freshwater sources currently unused.

A response team that includes researchers from several disciplines was organized to determine the best response to this problem. **Construct a concept map** that considers how decisions should be made during the different stages of the research process. This concept map should include who should be involved (i.e., relevant actors/stakeholders) and what types of information or data should be included at each of the following stages:

- Conceptualization of the problem
- Collection of data, information, and knowledge relevant to the issue
- Analysis and interpretation of data, information, and knowledge
- Use of data, information, and knowledge in reaching a decision about what to do

To aid you in the construction of the concept map, we have created a “scaffold” of these stages on the next page – add to these nodes to create your own unique concept map. Feel free to draw linkages between the nodes you construct to convey your perception of their relationships.

After you are complete, I will ask you a few questions about your concept map and more generally about your views on policy and research.

If you have any questions or confusion during this process, please feel free to ask.

**Case study II: Scientific evaluation and the ethical dimensions of invasive species management in Pascagoula, Mississippi.**

The coastal city of Pascagoula, Mississippi is considering a variety of management practices to respond to an influx of invasive locusts that were introduced to the region. Some policy makers and citizens think the city should take steps to eradicate the locusts. However, the specific impacts of the locusts are unknown, and there is no consensus about what steps should be taken. Some stakeholders believe that the city should use low-dosage pesticides to exterminate locusts in areas where locust calls seem to be harming the city’s economy. Others believe that these pesticides may pose unanticipated risks and that the city should instead explore regulating the household lighting that attracts the locusts or even relocating these businesses.

A response team that includes researchers from several disciplines was organized to determine the best response to this problem. **Construct a concept map** that considers how decisions should be made during the different stages of the research process. This concept map should include who should be involved (i.e., relevant actors/stakeholders) and what types of information or data should be included at each of the following stages:

- Conceptualization of the problem
- Collection of data, information, and knowledge relevant to the issue
- Analysis and interpretation of data, information, and knowledge
- Use of data, information, and knowledge in reaching a decision about what to do

To aid you in the construction of the concept map, we have created a “scaffold” of these stages on the next page – add to these nodes to create your own unique concept map. Feel free to draw linkages between the nodes you construct to convey your perception of their relationships.

After you are complete, I will ask you a few questions about your concept map and more generally about your views on policy and research.

If you have any questions or confusion during this process, please feel free to ask.
